# Molecular characterization of glucose-6-phosphate dehydrogenase deficiency in Jeddah, Kingdom of Saudi Arabia

**DOI:** 10.1186/1756-0500-4-436

**Published:** 2011-10-24

**Authors:** Soad K Al-Jaouni, Jummanah Jarullah, Essam Azhar, Kamran Moradkhani

**Affiliations:** 1 Hematology Research Lab, King Fahd Medical Research Centre, Faculty of Medicine, King Abdulaziz University, P.O. Box 80215, Jeddah 21589, Kingdom of Saudi Arabia; 2 Medical Molecular Virology Unit, King Fahd Medical Research Center, Faculty of Applied Medical Sciences, King Abdulaziz University, Jeddah, Kingdom of Saudi Arabia; 3 AP-HP, Hôpital H. Mondor-A. Chenevier, Service de Biochimie et Génétique, Créteil, 94000, France; 4 Inserm, U955, Créteil, 94000, France

## Abstract

**Background:**

The development of polymerase chain reaction (PCR)-based methods for the detection of known mutations has facilitated detecting specific red blood cell (RBC) enzyme deficiencies. We carried out a study on glucose-6-phosphate dehydrogenase (G6PD) deficient subjects in Jeddah to evaluate the molecular characteristics of this enzyme deficiency and the frequency of nucleotide1311 and IVS-XI-93 polymorphisms in the glucose-6-phosphate dehydrogenase gene.

**Results:**

A total of 1584 unrelated Saudis (984 neonates and 600 adults) were screened for glucose-6-phosphate dehydrogenase deficiency. The prevalence of glucose-6-phosphate dehydrogenase deficiency was 6.9% (n = 110). *G6PD Mediterranean *mutation was observed in 98 (89.1%) cases, *G6PD Aures *in 11 (10.0%) cases, and *G6PD Chatham *in 1 (0.9%) case. None of the samples showed *G6PD A‾ *mutation. Samples from 29 deficient subjects (25 males and 4 females) were examined for polymorphism. The association of two polymorphisms of exon/intron 11 (*c.1311T/IVS-XI-93C*) was observed in 14 (42.4%) of 33 chromosomes studied. This association was found in 9 (31.0%) carriers of *G6PD Mediterranean *and in 4 (13.8%) carriers of *G6PD Aures*.

**Conclusions:**

The majority of mutations were *G6PD Mediterranean*, followed by *G6PD Aures *and < 1% *G6PD Chatham*. We conclude that 1311T is a frequent polymorphism in subjects with *G6PD Mediterranean *and *Aures *variants in Jeddah.

## Background

Glucose-6-phosphate dehydrogenase (G6PD) deficiency is one of the most common enzymopathies, accounting for over 400 million cases worldwide [1-3]. Most cases of glucose-6-phosphate dehydrogenase deficiency occur in males [4]. Affected individuals are usually asymptomatic, and go through life without being aware of their deficiency. They are, however, at risk of having acute hemolytic crises in response to infection, eating fava beans, and to drugs having a high oxidation potential [5].

Based on biochemical properties, ethnic origin, and clinical presentation, more than 400 variants of glucose-6-phosphate dehydrogenase have been distinguished in the past. Characterization at the DNA level reduced this number to about 182; these are distributed among 4 classes based on activity and clinical manifestation [6]. In the Kingdom of Saudi Arabia, studies have described the prevalence of glucose-6-phosphate dehydrogenase deficiency and associated enzyme variants, with *G6PD Mediterranean *found to be the most common [7-10]. In the current study, 110 cases of glucose-6-phosphate dehydrogenase deficiency were studied at the molecular level to evaluate the molecular heterogeneity of this deficiency and the frequency of the silent C/T polymorphism at nt 1311 in glucose-6-phosphate dehydrogenase deficient subjects in the Jeddah area.

## Methods

Unrelated Saudi adults and neonates were screened for glucose-6-phosphate dehydrogenase deficiency. For adults, blood samples were collected from the general population; this included blood donors, university students, and health-care workers. All neonates delivered from February 2002 to June 2002 at King Abdulaziz University Hospital and King Fahd Armed Force Hospital in Jeddah were also screened for glucose-6-phosphate dehydrogenase deficiency. Consent was obtained from the participants or their parents prior to conducting the research. The study was approved by the Biomedical Ethical Research Committee of the Faculty of Medicine of King Abdulaziz University (Reference No. 530-11)

Red blood cell glucose-6-phosphate dehydrogenase activity was measured with quantitative kits that use Sigma glucose-6-phosphate dehydrogenase H control (Sigma Medical, USA). The kits use the chemical reaction described by Beutler [11], with NADPH production measured at 340 nm in kinetic mode (change in optical density per minute). Values < 4.6 U/g Hb were considered deficient [11,12].

Genomic DNA was extracted from peripheral blood leukocytes of the deficient subjects by standard methods (proteinase K digestion, phenol/isoamyl alcohol/chloroform extraction and ethanol precipitation) [11,13].

The most frequent glucose-6-phosphate dehydrogenase variants were determined by restriction fragment length polymorphism (RFLP). The *G6PD Aures *mutation is located in exon 3 (c.143T > C, p.Ile48Thr), *G6PD A‾ *mutations are located in exon 4 (c.202 G > A, p.Val68 > Met) and exon 5 (c.376A > G, p.Asn126Asp), and *G6PD Mediterranean *is in exon 6 (c.563C > T, p.Ser188Phe). Therefore, these DNA fragments were amplified using primers as listed in Table 1. For the reactions, 500 ng DNA were amplified on a GeneAmp^® ^PCR System 2700 thermal cycler (Applied BioSystems, Foster City, CA, USA) with 1.25 U AmpliTaq^® ^DNA Polymerase (Roche, New Jersey, NJ, USA) and 10 pmol of each primer, 0.2 mM of each dNTP, and1.5 mM MgCl_2_. The final buffer comprised 16.6 mM (NH_4_)_2_SO_4_, 67 mM Tris-Cl, pH 8.8, 1.5 mM MgCl_2_, 67 mM Na_2_EDTA, pH 8.0, 70 μg BSA, and 10 mM β-mercaptoethanol. An initial denaturation step at 94°C for 5 minutes was followed by 35 cycles of denaturation at 94°C for 10 seconds, annealing at 61°C for 30 seconds, and extension at 72°C for 30 seconds. A final extension at 72°C for 7 minutes completed the cycling reaction. For RFLP studies, 18 μl of each PCR product was restricted according to the manufacturer's recommendation with 20 IU of each corresponding enzyme. Exon 3 was cleaved with BglII, exon 4 with NlaIII, exon 5 with FokI, and exon 6 with MboII. Ten microliters of the digestion products were size fractionated by electrophoresis through polyacrylamide gels (6%), stained with ethidium bromide (0.25 μg/ml), and analyzed by UV transillumination.

**Table 1:** Primers used for identification of glucose-6-phosphate dehydrogenase defects^a^

Analysis	Analyzed Region	Forward 5'→3'	Reverse 5'→3'
RFLP Analysis (Tm = 61°C)	Exon 3	GTGGAGGATGATGTATGTAGGT	AGGGCAGGGCACAGCTGTAA
	
	Exon 4	TACAGTCGTGCCCTGCCCT	CCGAAGCTGGCCATGCTGG
	
	Exon 5	CTGTGTGTGTGTCTGTCTGTC	GGAGGGCAACGGCAAGCCTT
	
	Exon 6	GCAGCTGTGATCCTCACTCC	GCAAGGTGGAGGAACTGACC

Gene sequencing (Tm = 56°C)	Exons 1, 2	AAGCTCGGTAATGATAAGCACGC	TGGAGCAGGCACTTCCTGG
	
	Exons 3, 4, 5	GTGGAGGATGATGTATGTAGGT	GGAGGGCAACGGCAAGCCTT
	
	Exons 6, 7, 8	AGAGGGTCATCTGGGAACACAA	CCTGGGACATGACAACTTGGG
	
	Exons 9, 10, 11, 12, 13	TCTGTGGCCACAGTCATCCC	CGCCCTCCTCCTTCCTTCTG

Abbreviations: A, alanine; C, cysteine; G, glycine; RFLP, restriction fragment length polymorphism; Tm, Anealing temperature; T, threonine

^a^Adapted from Moradkhani et al.[14]

For molecular characterization of the remaining glucose-6-phosphate dehydrogenase deficient subjects and also for evaluating the frequency of nucleotide 1311C > T polymorphism, the glucose-6-phosphate dehydrogenase gene was entirely sequenced. Samples from glucose-6-phosphate dehydrogenase deficient patients were sent to the Department of Biochemistry and Genetics, Henri Mondor University Hospital, Creteil, France for gene sequencing. Different DNA fragments were amplified using primers as listed in Table 1. The amplification reaction was performed using 10% (v/v) dimethylsulphoxide (DMSO) with 35 cycles of 1 minute denaturation at 94°C, 1 minute annealing at 56°C, and 1 minute extension at 72°C, followed by a final extension of 7 minute at 72°C. Nucleotide sequencing of the PCR products was performed with the forward and reverse primers using the BigDye^® ^Terminator v3.1 Sequencing Kit (Applied BioSystems) and analyzed on an automated sequencer (ABI PRISM^® ^3100 Genetic Analyzer; Applied BioSystems) [3,14,15].

## Results

A total of 1584 Saudis (984 neonates and 600 adults) were screened for glucose-6-phosphate dehydrogenase deficiency. One hundred and ten subjects (6.9%), including 42 adults (36 males and 6 females) and 68 neonates (58 males and 10 females) were deficient. *G6PD Mediterranean *mutation was observed in 98 (89.1%) cases, *G6PD Aures *in 11 (10.0%) cases, and *G6PD Chatham *in 1 (0.9%) case. None of the samples showed *G6PD A‾ *mutation (Table 2). Samples from 29 deficient subjects (25 males and 4 females) were examined for polymorphism. The nucleotide *1311T *(p.Tyr437Tyr) polymorphism was observed in 14 (42.4%) of 33 chromosomes studied (25 males and 4 females), and it was associated with *G6PD Mediterranean *(n = 9; 31.0%) and *G6PD Aures *(n = 4; 13.8%) (Table 2).

**Table 2:** Summary of mutations detected in 110 deficient Saudi patients in Jeddah and the frequency of 1311T and IVS-XI-93C polymorphisms.

G6PD Variants	Mutation	Amplified Exon/Intron	Number of Patients and Percentage [n (%)]
*Mediterranean*	*c.563 C > T*	6	98 (89.1%)

*Aures*	*c.143 T > C*	3	11 (10.0%)

*G6PD A^¯^*	*c.202 G > A*	4	0 (0%)
	
	*c.376 A > G*	5	0 (0%)

*Chatham*	*c.1003 G > A*	9	1 (0.9%)

Polymorphism (33 chromosomes of 25 males and 4 females)	*c. 1311T*	11	14 (42.4%)
	
	*IVS-XI-93C*	Intron 11	21 (63.6)
	
	*1311T/IVS-XI-93C*	Exon/intron 11	14 (42.4%)

Abbreviations: A, alanine; C, cysteine; G, glycine; G6PD, glucose-6-phosphate dehydrogenase; T, threonine

## Discussion

The availability of molecular studies by PCR and glucose-6-phosphate dehydrogenase gene sequencing allows accurate diagnosis and characterization of glucose-6-phosphate dehydrogenase deficiency. In this study, we characterized the molecular defects of the glucose-6-phosphate dehydrogenase gene in deficient patients living in Jeddah. We identified 3 variants; *G6PD Mediterranean*, *G6PD Aures *and *G6PD Chatham *(Table 2).

Most of the patients (89.1%) in this study had the *G6PD Mediterranean *mutation. Similarly, other authors found a high prevalence of *G6PD Mediterranean *among glucose-6-phosphate dehydrogenase deficient Saudis in a study conducted in Jeddah [8,9]. These studies, however, reported a lower frequency compared with our study; 51.1% and 38.1%, respectively. In one report evaluating the frequency of glucose-6-phosphate dehydrogenase mutation in 47 patients in Jeddah, 96% of the patients showed C > T substitution at codon 188 of the glucose-6-phosphate dehydrogenase gene, characteristic of the Mediterranean type [8]. Another study from the Al-Hassa and Al-Qatif areas of Saudi Arabia's Eastern Province also showed *G6PD Mediterranean *as the most frequent variant, with a prevalence of 45.9% and 36.5% for these 2 areas [10]. Studies conducted in several other areas of the Persian Gulf region have also reported a high prevalence of the Mediterranean variant; 55.5% in the Al-Ain District of United Arab Emirates [16], 72.9% in Kuwait [17], 65.2% in Oman [18], and 72.8% in Zanjan Province in Iran [19].

*G6PD Aures *was first described in Saudi Arabia by Niazi et al. [20] in 1996. In the current study we identified *G6PD Aures *in 10% of the cases (Table 2). Similar results were reported from Taif, in the Makkah province of Saudi Arabia, with 12.3% of 49 deficient Saudis having *G6PD Aures *[7]. The prevalence was slightly higher in the United Arab Emirates (16.6%) [10] and much lower in Kuwait (1.4%) [17].

The *G6PD A^¯ ^*variant was absent in our sample. Warsy and El-Hazmi [21] identified the *G6PD A^¯ ^*in a study conducted in several provinces in Saudi Arabia. The central province had the lowest frequency (0.0325 in males and 0.0242 in females for *A^+^*, 0.0074 in males and 0.0009 in females for *A^¯^*). This report primed us to authenticate the data by sequencing all the samples. However a larger sample size is needed for the verification of absence of *G6PD A^¯ ^*mutation.

The low prevalence (0.9%) of *G6PD Chatham *mutation in this study was less than that reported in another study [7] from Taif, where the authors found a prevalence of 4.1%. The lower prevalence observed in our study could be due to the fact that we had a larger sample size (110) compared with 49 in their study.

In this study, the association of two polymorphisms, a silent C/T at nt 1311 (p.Tyr437Tyr) and T/C at nt IVS-XI-93, was studied by direct sequencing of exon and intron 11 of the glucose-6-phosphate dehydrogenase gene. The *c.1311T/IVS-XI-93C *polymorphism was identified in 42.2% of 33 chromosomes studied. This result is comparable to that obtained from studies conducted in Bahraini and Pakistani populations [22]. On the other hand, Kurdi-Haidar et al. [23] reported the frequency of the *c.1311T/IVS-XI-93C *polymorphism in only 14% of the subjects in a study conducted in Saudi Arabia. This can be explained by the low number of X chromosomes studied by the authors. The *1311T *polymorphism is frequently observed in Indian and Middle East populations [24]. Conversely, it is less frequent in American and non Mediterranean European countries [22,24].

The presence of the dual mutation (*c.1311T/IVS-XI-93C*) has been proposed to be linked to glucose-6-phosphate dehydrogenase deficiency. This association was frequently observed with *G6PD Mediterranean *and *G6PD Aures *[25,26].

## Conclusions

Molecular analysis of glucose-6-phosphate dehydrogenase deficiency in this study conducted in Jeddah revealed a higher prevalence of *G6PD Mediterranean *(89.1%) and *Aures *(10.0%) compared with other studies from Saudi Arabia. The absence of the African variant suggests further studies with a larger sample size are warranted. The nucleotide 1311T is a frequent polymorphism observed in Saudi Arabia.

## Competing interests

The authors declare that they have no competing interests.

## Authors' contributions

SKJ, as the principal investigator, designed and wrote the proposal for the study, and submitted it to the research committee of King Abdulaziz University in April 2006. EA helped supervise gel documentation and sequencing of glucose-6-phosphate dehydrogenase in his laboratory. JJ performed the molecular procedure with the assistance of SKJ. KM conducted the gene sequence analysis of the 42 samples. All authors contributed to writing the manuscript.

## References

[B1] Beutler E Glucose-6-phosphate dehydrogenase deficiency: a historical perspective Blood 2008 111 1 16 24 10.1182/blood-2007-04-077412 18156501

[B2] Vulliamy TI Beutlerand E Luzzatto L Variants of glucose-6-phosphate dehydrogenase are due to missense mutations spread throughout the coding region of the gene Hum Mutat 1993 2 159 163 10.1002/humu.1380020302 8364584

[B3] Luzzatto L Mehta A Vulliamy TJ Schriver CR, Beaudet AL, Sly WS, Valle D Glucose 6-phosphate dehydrogenase deficiency The Metabolic and Molecular Bases of Inherited Disease 2001 8 McGraw-Hill. New York 4517 4553

[B4] Laosombata V Sattayasevanaa B Janejidamaib W Viprakasitic V Shirakawad T Nishiyamad K Matsud M Molecular heterogeneity of glucose-6-phosphate dehydrogenase (G6PD) variants in South of Thailand and identification of novel variant(G6PD) SongIanagarind Blood Cells, Molecules and Disease 2005 34 191 196 10.1016/j.bcmd.2004.11.001 15727905

[B5] Beutler E G6PD: Population genetics and clinical manifestations Blood Reviews 1996 10 45 52 10.1016/S0268-960X(96)90019-3 8861278

[B6] Beutler E G6PD deficiency Blood 1994 84 3613 3636 7949118

[B7] Mohamed Magdy M Rahmain Abd UI Humiany El Molecular Characterization of New Variants of G6PD deficiency Gene Isolated in western province of Saudi Arabia causing Haemolytic Anemia Pakistan Journal of Biological sciences 2006 9 9 1605 1616

[B8] Gari MA Chaudhary AG Al-Qahtani MH Abuzenadah AM Waseem A Banni H Al-Sayes FM Al-Harbi A Lary S Frequency of Mediterranean mutation among a group of Saudi G6PD patients in Western region-Jeddah Int J Lab Hematol 2010 32 17 21 10.1111/j.1751-553X.2008.01108.x 20447239

[B9] Al Jaouni SK Qari M Ashankyty I Al-Mahayawi S Jarullah J Glucose 6 Phosphate Dehydrogenase Deficiency Correlation between Genotype and Phenotype JKAU Med Sci 2007 14 2 3 13

[B10] Al-Ali AK Common G6PD variant from Saudi population and its prevalence Ann Saudi Med 1996 16 6 654 6 17429252 10.5144/0256-4947.1996.654

[B11] Beutler E Blume KG Kaplan C Lohr W Ramot B Valetinine WN International committee for standardization in Hematology Recommended screening test for glucose-6-phosphate dehydrogenase (G-6-PD) Br J Haematol 1979 43 469 477 10.1111/j.1365-2141.1979.tb03775.x 497122

[B12] Echler G Determination of glucose-6-phosphate dehydrogenase Am J Med Technol 1983 49 259 6846390

[B13] Maniatis T Fritsch EF Sambrook J Molecular Cloning: A Laboratory Manual Cold Spring Harbour Lab NY 1982

[B14] Moradkhani K Bahuau M Prehu C Martin N Bimet C Galateros F Wajcman H A rare G6PD variant (c.383T > G; p.128Leu > Arg) with a molecular pathophysiological mechanism similar to that G6PD A- (68Val > Met, 126Asn > Asp) Blood Cells Mol Dis 2009 3 3 226 229 10.1016/j.bcmd.2009.05.007 19632868

[B15] Nafa K Regis A Osmani N Baghli L Benabadji M Kaplan JC Vulliamy TJ Luzzatto L G6PD Aureus: a new mutation (48 Ile-- > Thr) causing mild G6PD deficiency is associated with favism Hum Mol Genet 1993 2 1 81 82 10.1093/hmg/2.1.81 8490627

[B16] Bayoumi RA Nur-E-Kamal MS Tadayyon M Mohamed KK Mahboob BH Qureshi MM Lakhani MS Awaad MO Kaeda J Vulliamy TJ Luzzatto L Molecular characterization of erythrocyte glucose-6-phosphate dehydrogenase deficiency in Al-Ain District, United Arab Emirates Hum Hered 1996 46 3 136 141 10.1159/000154342 8860007

[B17] Samilchuk E Al-Suliman I Usanga E Al Awadi S Glucose-6-phosphate dehydrogenase (G6PD) mutations and UDP-glucuronosyltransferase promoter polymorphism among G6PD deficient Kuwaitis Blood Cells Mol Dis 2003 31 2 201 205 10.1016/S1079-9796(03)00125-6 12972027

[B18] Daar S Vulliamy TJ Kaeda J Mason PJ Luzzatto L Molecular characterization of G6PD deficiency in Oman Hum Hered 1996 46 3 172 176 10.1159/000154348 8860013

[B19] Mortazavi Y Mirzamohammadi F Teremahi Ardestani M Miri-Moghadam E Vulliamy TJ Glucose 6-phosphate dehydrogenase deficiency in Tehran, Zanjan and Sistan-Balouchestan provinces: prevalence and frequency of Mediterranean variant of G6PD Iran J Biotechnol 2010 8 4 229 233

[B20] Niazi GA Adeyokunnu A Westwood B Beutler E Neonatal jaundice in Saudi newborns with G6PD Aures Ann Trop Paediatr 1996 16 1 33 7 8787363 10.1080/02724936.1996.11747801

[B21] Warsy AS El-Hazmi MAF G6PD Deficiency, Distribution and Variants in Saudi Arabia: An Overview Ann Saudi Med 2001 21 3-4 174 177 17264545 10.5144/0256-4947.2001.174

[B22] Moiz B Nasir A Moatter T Naqvi ZA Khurshid M Population study of 1311 C/T polymorphism of Glucose 6 Phosphate Dehydrogenase gene in Pakistan-an analysis of 715 X-chromosomes BMC Genet 2009 10 41 19640310 10.1186/1471-2156-10-41 PMC2725355

[B23] Kurdi-Haidar B Mason PJ Berrebi A Ankra-Badu G al-Ali A Oppenheim A Luzzatto L Origin and spread of the glucose-6-phosphate dehydrogenase variant (G6PD-Mediterranean) in the Middle East Am J Hum Genet 1990 47 6 1013 1019 1978555 PMC1683892

[B24] Mortazavi Y Chopra R Gordon-Smith EC Rutherford TR Frequency of the G6PD nt 1311 C/T polymorphism in English and Iranian populations: relevance to studies of X chromosome inactivation J Med Genet 1997 34 12 1028 1029 10.1136/jmg.34.12.1028 9429150 PMC1051159

[B25] Yang Z Chu J Ban G Huang X Xu S Li M The genotype analysis of glucose-6-phosphate dehydrogenase deficiency in Yunnan province Zhonghua Yi Xue Yi Chuan Xue Za Zhi 2001 18 4 259 63 11484161

[B26] Amini F Ismail E Zilfalil BA Prevalence and molecular study of G6PD deficiency in Malaysian Orang Asli Intern Med J 2011 41 4 351 3 10.1111/j.1445-5994.2011.02456.x 21507164

